# Design of a Broadband Electrical Impedance Matching Network for Piezoelectric Ultrasound Transducers Based on a Genetic Algorithm

**DOI:** 10.3390/s140406828

**Published:** 2014-04-16

**Authors:** Jianfei An, Kezhu Song, Shuangxi Zhang, Junfeng Yang, Ping Cao

**Affiliations:** Department of Modern Physics, University of Science and Technology of China, and Key Laboratory of Technologies of Particle Detection & Electronics, Chinese Academy of Science, Hefei 230026, China; E-Mails: ajf@mail.ustc.edu.cn (J.A.); shuangxiz@163.com (S.Z.); yangjf@ustc.edu.cn (J.Y.); caop@ustc.edu.cn (P.C.)

**Keywords:** broadband piezoelectric transducer, genetic algorithm, electrical impedance matching network, automated circuit design

## Abstract

An improved method based on a genetic algorithm (GA) is developed to design a broadband electrical impedance matching network for piezoelectric ultrasound transducer. A key feature of the new method is that it can optimize both the topology of the matching network and perform optimization on the components. The main idea of this method is to find the optimal matching network in a set of candidate topologies. Some successful experiences of classical algorithms are absorbed to limit the size of the set of candidate topologies and greatly simplify the calculation process. Both binary-coded GA and real-coded GA are used for topology optimization and components optimization, respectively. Some calculation strategies, such as elitist strategy and clearing niche method, are adopted to make sure that the algorithm can converge to the global optimal result. Simulation and experimental results prove that matching networks with better performance might be achieved by this improved method.

## 1. Introduction

**Figure 1.**
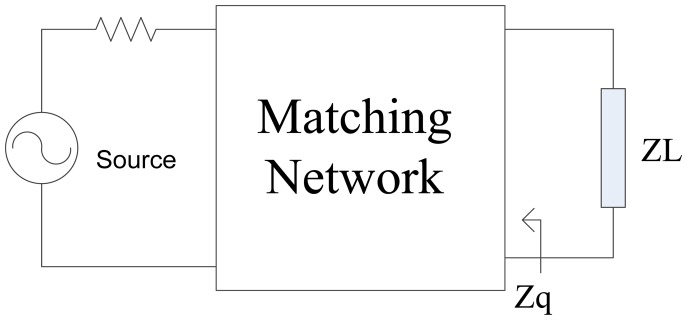
The matching network is inserted between the source and load.

Piezoelectric transducers have been widely applied in different fields [1,2]. Most traditional piezoelectric transducers are narrowband, and can only produce large scale vibrations within a narrow range near their resonant frequency. However more and more applications need transducers to work within rather wide ranges. A number of papers have recently focused on how to design various types of broadband piezoelectric transducers [3,4]. In the oil exploration logging industry, some leading companies such as Schlumberger have designed broadband transducers working within the range of 200 kHz–700 kHz and have applied them in production. Like narrowband transducers, the equivalent impedance of broadband transducers is capacitive too. In most cases, the excitation source and the transducer are impedance mismatched. If no electrical impedance matching network (EIMN) is adopted, the power reflected from the transducer will reduce the efficiency of the source and, as the condition worsens, even cause permanent damage to components. For a narrowband piezoelectric transducer, a suitable inductance would be enough in an EIMN. As for a broadband piezoelectric transducer (BPT), however, methods of designing EIMNs are usually complicated and hard to understand. Although different methods [5,6] of designing EIMNs have been developed, it is still meaningful to find a method that is easy to realize in practice and has good performance at the same time. In this paper, a brief review about methods of designing EIMNs is made first. Second, a method based on a genetic algorithm (GA) developed in this paper is described in detail. Third, simulation results of several examples are given to confirm the effectiveness of the method. Last, this paper is summarized briefly. A general purpose of designing EIMNs is to make the signal's energy transfer from source to load as much as possible within a broad frequency range. It is realized by means of inserting a lossless two ports matching network between the source and the load as shown in Figure 1.

The performance of the EIMN in Figure 1 can be evaluated by a parameter called transducer power gain (TPG) which is defined by Equation (1):

(1) $$\text{TPG}=\frac{4R_{Q}R_{L}}{{\left(R_{Q}+R_{L}\right)}^{2}+{\left(X_{Q}+X_{L}\right)}^{2}}$$

where Z_L_ = R_L_ + jX_L_ and Z_Q_ = R_Q_ + jX_Q_. Z_Q_ is the impedance seen from Z_L_ to the matching network. Through simple calculation, the value of TPG is between 0 and 1. A common purpose of the matching network is to make the TPG as big as possible within a specified frequency range.

There are mainly two types of broadband impedance matching methods: the analytical method and the CAD method. The analytical method started from Bode's theoretical research on impedance matching [7]. He studied the matching problem of RC parallel loads and the gain-bandwidth limitation of the problem was obtained. Fano [8] developed Bode's theory and solved the matching problem of any kind of load in a more general way. Youla [9] reconsidered this problem based on complex normalization theory. If the analytical form of a load is known, a matching network can be designed using Fano and Youla's theory. Although the analytical method can solve any impedance matching problem if the analytical form of the load is known, the method is very complicated and besides, most of the time the analytical form of the load is unknown. These shortcomings limit the theory's practice in engineering.

In order to overcome the limitations of analytical theory, Carlin [6] first developed the CAD method, namely the real frequency technique (RFT), for solving single matching problem. Using measured data, a two-port lossless matching network between any kind of load and resistive source can be calculated by RFT without having to know analytical form of the load or predefining the structure of the matching network. Although complex derivation is avoided, results achieved by RFT are dependant on the initial values. In 1982, Yarman [10] proposed a simplified real frequency technique (SRFT). Compared to RFT, the Hilbert transform is not necessary in SRFT, but polynomial explicit factorization and solving linear equations are still needed. In engineering, RFT or SRFT have been widely adopted in designing broadband EIMNs [11,12]. However, there are still some shortcomings such as the fact they can only synthesize the topology of LC ladder structure. Dedbeu [13] has pointed out that an EIMN that has resonant units might have better performance than an EIMN of LC ladder structure, but it is not mentioned that how many resonant units are needed and the locations of the resonant units in network is determined by a rather arbitrary way.

Another different CAD method is the recursive stochastic equalization (RSE) method [14]. Based on an initially imposed topology, TPG is obtained as the objective function which has a function relationship with frequency and the impedance of the matching network. A stochastic Gauss-Newton algorithm followed by a limited random search is used to find the optimal value of the objective function. The final result of RSE method is not dependant on the initial parameters of the objective function, however, it is difficult to decide what kind of topology is appropriate, especially when the EIMN topology is complex.

A relatively simple method commonly applied in RF analog circuit design is based on Smith charts [5]. It assumes that the bandwidth has an inverse relationship with the quality factor Q. Most likely, it is true because the impedance changes within a small range and has only one peak within its working frequency range, but this is not the case when the method is applied to BPT. The impedance of BPT usually has multiple peak values and changes greatly in which case the inverse relationship between bandwidth and quality factor is not true anymore.

From the above discussion, it can be concluded that EIMNs synthesized by current typical methods still might have room for improvement. This paper puts forward to a method of designing broadband EIMNs based on a GA. This method can find the optimal topology as well as the components values automatically within a set of predefined topology. The definition of the set is very important to the success of the algorithm and is selected based on the experiences of current methods.

The GA is a global searching algorithm that has been widely used in many areas [15,16]. In circuit design field, the GA can be used to explore wider design space and obtain novel and better results without relying on *a priori* knowledge and rules. Compared with digital circuits, the structure, component types and parameters of analog circuits are complex. Huge operations will be inevitable and rise exponentially with the circuit scale if a GA is adopted to design analog circuits. A typical analog circuit evolution method proposed by Koza [17] is called genetic programming (GP). Because of its refusal to use prior knowledge and the pursuit of a rich circuit structure and parameters, it will take a few days to design a cell circuit such as low pass filter by GP, even using a high performance parallel computer cluster. To simplify the computation process, Lohn [18] proposed a new circuit presentation method called trail encoding. Although the computation is reduced significantly, a network of high performance parallel workstations is still needed. In order to reduce the computational complexity so that the algorithm can run on a laptop, the set of the candidate topologies must be limited.

## 2. Design of Broadband EIMN Based On GA

### 2.1. Overview of the Algorithm

The idea of the GA-based method we present here is to find a way in which not only both the network topology and the component values can be optimized, but also the algorithm doesn't need a lot of computing resources so that it can run on a laptop. To reduce the computing resources needs and make sure the performance of the method is good, prior experiences are referenced to limit the number of candidate topologies.

**Figure 2.**
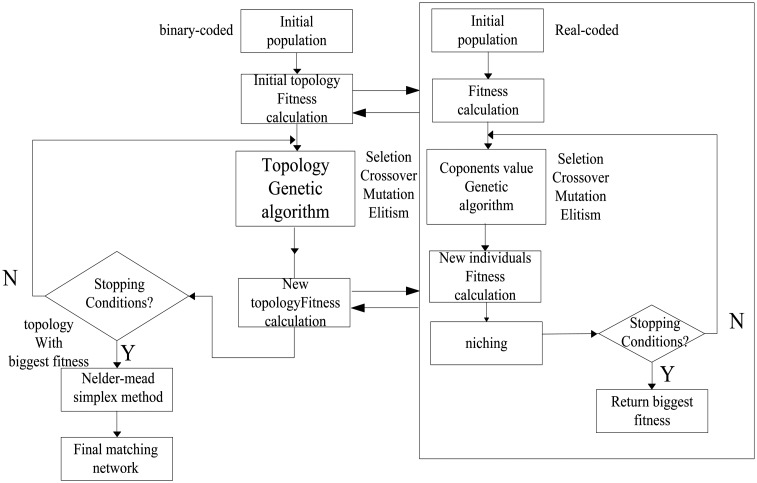
Overview of the new method based on GA.

The execution process of the method is shown in Figure 2. The topology optimization and the value optimization are separated. A binary-coded GA is adopted to evolve the topology and a real-coded GA is followed to determine the components values and calculate the TPG of the topology.

The whole process of the calculation is divided into two parts. First, the binary coded GA is applied to generate network topologies. In the GA, the performance of every individual is measured by a parameter called fitness. In this paper, the TPG of the topology is chosen to be its fitness. To calculate the TPG of a topology, the components values have to be assigned first. Here, a real-coded GA is applied to find the optimal components values of every topology generated by the binary coded GA, and then, the fitness of the topology can be calculated easily.

**Figure 3.**
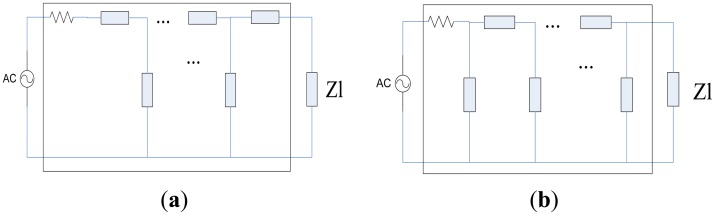
(**a**) T type of matching network and (**b**) π type of matching network.

To improve the ratio of effectiveness of new topology and reduce computation time, the matching network is assumed to have the ladder-like structure that is shown in Figure 3. The types of Z1, Z2, … Zn could be one of the four options which are L, C, parallel LC and serial LC and they are determined automatically by the algorithm.

The reason why the ladder-like structure is chosen is that it has been proved to be effective for most EIMN problems and some popular methods are based on this structure [5,6]. Another reason is that it can assure that most of the new networks generated by the algorithm are valid and can be evaluated easily which can reduce the computation time.

The number of branches in the structure is pre-specified, but it is not determined arbitrarily. The way the number is chosen is based on the conclusion that, for a specified load, if a matching network with LC ladder structure is applied, there will be a critical number of the branches. When the number of branches is less than the critical value, the more branches are, the better the performance of the EIMN is, and when it outnumbers the value, the performance is nearly invariable or even declines as the number increases. The critical number of branches of the LC ladder structure is specified to be the number of Zn in our algorithm. In part 3, an example will be used to prove that it is a feasible idea.

### 2.2. Topology Optimization Method

The basic flow of GA [19] used for topology optimization can be represented as SGA = (C, F, P, M, φ, Г, Ψ, T;, in which *C* is the form the matching network topology is encoded; *F* is the fitness function of a topology; *P* is the initial population of GA; *M* is the number of individuals in the population; Φ, i and Ψ represent selection, crossover and mutation operator respectively and T is the termination condition of GA.

In the algorithm, the matching network topologies are binary-encoded. Each branch is represented using two binary bits, which is shown below.


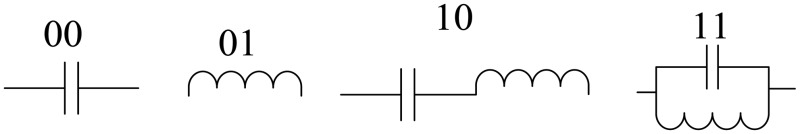


The fitness function we use here is shown in Equation (2) in which TPG (ω) is shown in Equation (1):

(2) fitness=min(TPG(ω))

Each topology of the initial population is created randomly and the population size we use here is 120. Parents are selected by tournament [19] in three randomly-chosen individuals. Uniform crossover [19] is used as crossover operator by which each gene of new individual is randomly chosen in two parents. The probability of mutation [19] we use here is 0.1. The evolution will stop when the generation exceeds the maximum value *g*_max_ or a fitness value is bigger than a specified figure. The new individual generated in each generation is different from all individuals in the population. An elitist strategy [19] is adopted in that the individual with the best fitness in current generation will be reproduced to the next generation directly.

### 2.3. Values Optimization Method

To calculate the fitness of a topology, the components values of the topology need to be determined first. A real-coded genetic algorithm (RCGA) [20] is adopted to perform optimization on the components values. Although based on the same theory, some operators in RCGA are different from those in BCGA. A niching method [21] and the elitist strategy are adopted in combination to make sure the algorithm can find the global optimization solution of a multimodal function.

In a practical transducer excitation circuit, a pulse transformer is needed. The effect of the transducer is: changing voltage, transforming impedance and blocking DC. In our method, the parameter of the transformer is determined by means of finding the optimal source resistance Rg'. The relation between Rg' and real source resistance Rg is: Rg' = n^2^ × Rg. The parameter n is realized by a transformer: 
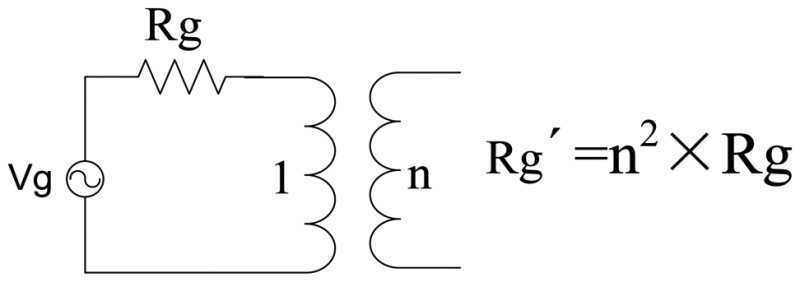


The commonly used stochastic universal sampling (SUS) [19] is selected to be the selector operator. BLX-a crossover [20] is chosen as the crossover operator. If C_1_ = (C_1_^1^,…,C_n_^1^) and C_2_ = (C_1_^2^,…,C_n_^2^) are two individuals which are selected to apply crossover operator to them, an offspring H = (h_1_,…, h_i_,…, h_n_) is generated where h_i_ is randomly chosen within the range of [C_min_ – I × a, C_max_ + I × a], C_max =_ max[C_i_^1^, C_i_^2^], C_min =_ min[C_i_^1^, C_i_^2^], I = C_max_ − C_min_ and a = 0.3.

Nonuniform mutation [20] is used as the mutation operator. Suppose C_i_ ε[a_i_,b_i_] is a gene to be muted. if *t* is a generation, *g*_max_is the maximum number of generations and τ is randomly chosen to be 1 or 0, then:

(3) $$C_{i}^{\prime }=\{\begin{array}{cc} C_{i}+\Delta (t,b_{i}-C_{i}) & if\tau =0\\ C_{i}-\Delta (t,C_{i}-b_{i}) & if\tau =1 \end{array}$$

and $\Delta (\text{t},\text{y})=\text{y}(1-r^{{\left(1-\frac{t}{g_{\text{max}}}\right)}^{b}})$. Parameter b is chosen by the user and we set b equal to 5 here. When GA is applied to optimize a multimodal function, it tends to converge to the local optimal values. To overcome this shortcoming, a clearing niching method is adopted [21]. In this method, the distance between two individuals is defined first, which is the Euclidean distance in our algorithm. The capacity of a niche is defined which means that within the predefined range of an individual, only a limited number of individuals that have bigger fitness than others are effective, while other individuals' fitness are cleared to zero. TPG is also used as the fitness of RCGA. When the algorithm finishes evolution, the genes in the chromosome that has the biggest fitness are assigned to the components of the specified topology.

Another shortcoming of the GA is that the efficiency will decrease markedly and be difficult to achieve the optimal solution when the algorithm is close to the optimal value. In order to solve this problem, a local optimization algorithm called the *Nelder-Mead* simplex algorithm (NMSA) [22] is adopted as a complement to RCGA. There is no need to put the NMSA in the iterative process because NMSA only modifies the result of RCGA slightly, so it has no decisive impact on the selection of the EIMN topology.

## 3. Simulation and Test

In this part, two examples are presented to prove the effectiveness of the algorithm.

### 3.1. EIMN of the Fano LCR Load

In this part, a LCR load is used first to testify the correctness of the method in Section 2. The reason why the example is used here is that Fano, Carlin and Dedieu have all used it to verify their results and it is convincing and comparable to verify the performance of a new method by calculating the matching network of the commonly used load [6,8,14]. It is a representative example that has been calculated by variable algorithms. By selecting this example, we can compare our solution with the results provided by other approaches. The load consists of a 1 Ω resistor in parallel with a 1.2 F capacitor and a 2.3 H inductor. The gain needs to be maximized within the range [0, 1] rad/s.

**Figure 4.**
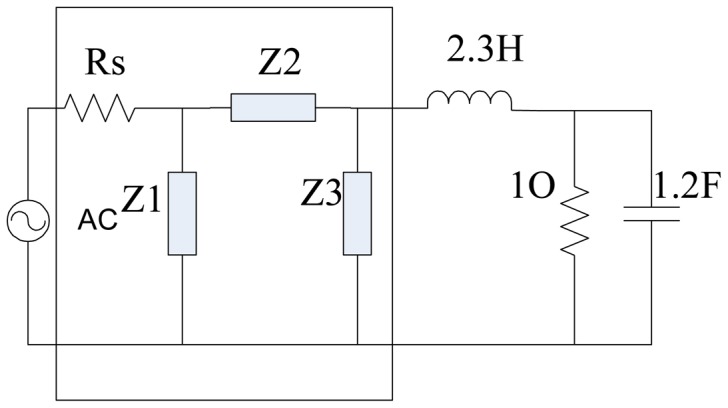
Initial EIMN topology of GA.

The EIMN topology of the LCR load tested by RFT and RSE is the same as the Figure 3 in [14]. The initial EIMN topology of our solution is shown in Figure 4. It can be concluded that because the frequency range is low-pass, there is no resonant unit in the topology achieved by our method and after optimization is over, it achieves the same topology as [14].

**Figure 5.**
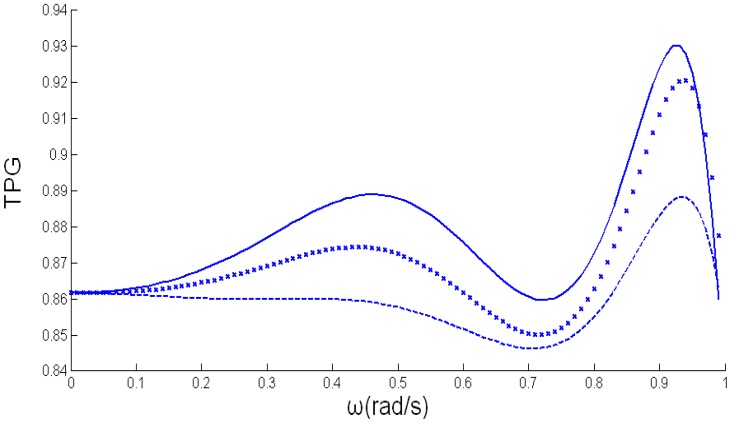
The TPG of the EIMNs: — GA; *RSE; --- RFT.

The components' values and the minimum of TPG obtained by the GA, RFT and RSE are shown in Table 1 [14]. The comparisons of TPGs within the designated band obtained by the three methods are shown in Figure 5. We can see that the Nelder-Mead simplex algorithm is just a refinement of the GA. Although the same topology is obtained, the T_min_ achieved by our method is a little better than by the results obtained RSE and RFT. These results have proved the validity of our method.

### 3.2. Broadband EIMN of A Piezoelectric Ultrasound Transducer Array

#### 3.2.1. Design and Simulation

In the oil exploration field, high-power broadband ultrasound transducers are widely used to detect the status of drilling pipes. Sometimes, a transducer array that includes several transducers working in different frequency bands is needed to expand frequency bandwidth.

**Figure 6.**
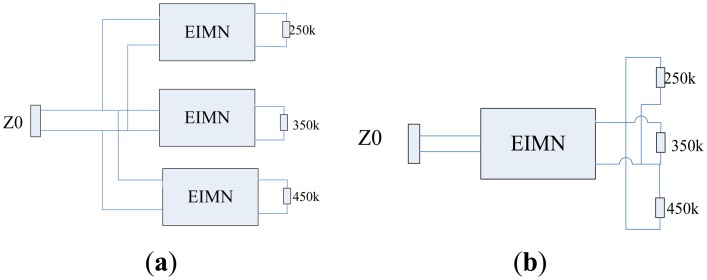
Different methods for matching the transducer array.

As is shown in Figure 6, a transducer array of which the working frequency is 200 kHz–500 kHz includes three high-power transducers centered at 250 kHz, 350 kHz and 450 kHz respectively.

The array can be matched by two different methods. In Figure 6a, each transducer is matched by an EIMN respectively. Because every transducer works within a narrower band, each EIMN can be realized by fewer components and high performance can be achieved. Compared to the complex structure in Figure 6a, the structure shown in Figure 6b is quite simple and all transducers are matched by a single EIMN. Because the EIMN in Figure 6b need to work within a wide band, more components will be needed. In this part, only the EIMN in Figure 6b will be designed and tested.

**Figure 7.**
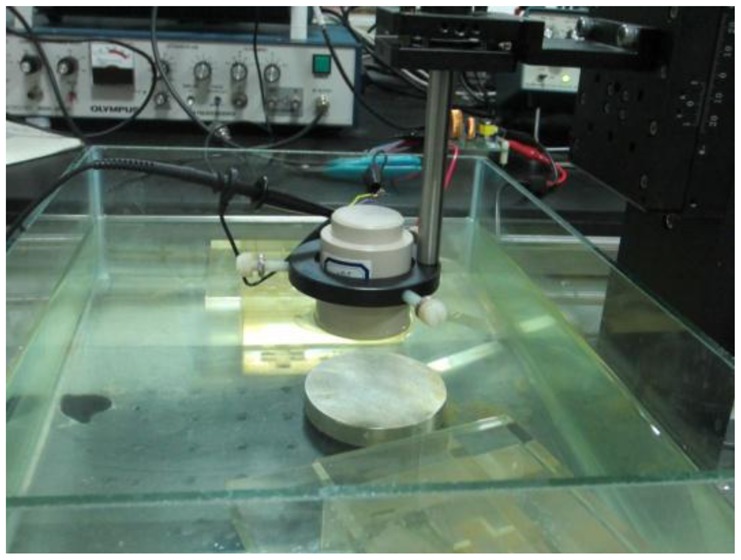
Impedance measurement of the transducer array.

To design the EIMN, the impedance of all transducers needs to be measured first. A HP4294A precise impedance analyzer has been used to complete the measurements. To get correct results, we must make sure that the transmitting surface of the transducer is under the surface of water during the measurements, as is shown in Figure 7.

**Figure 8.**
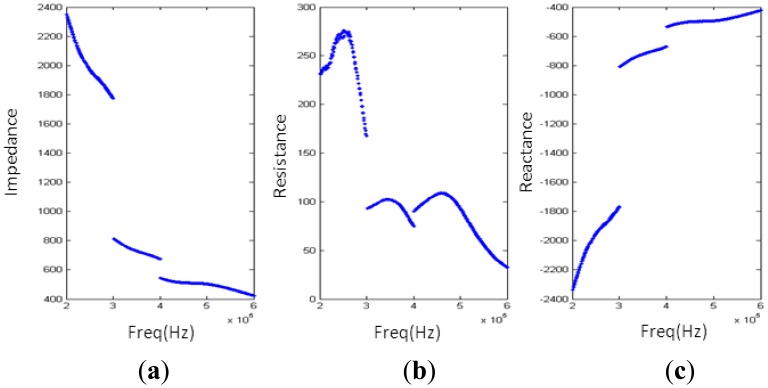
Results of impedance measurement: (**a**) impedance; (**b**) resistance; (**c**) reactance.

**Figure 9.**
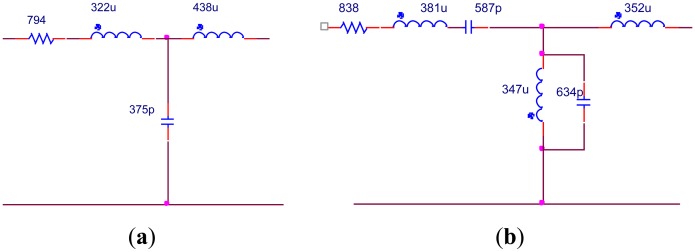
Results of two algorithms: (a) RFT; (b) algorithm introduced in Section 2.

**Figure 10.**
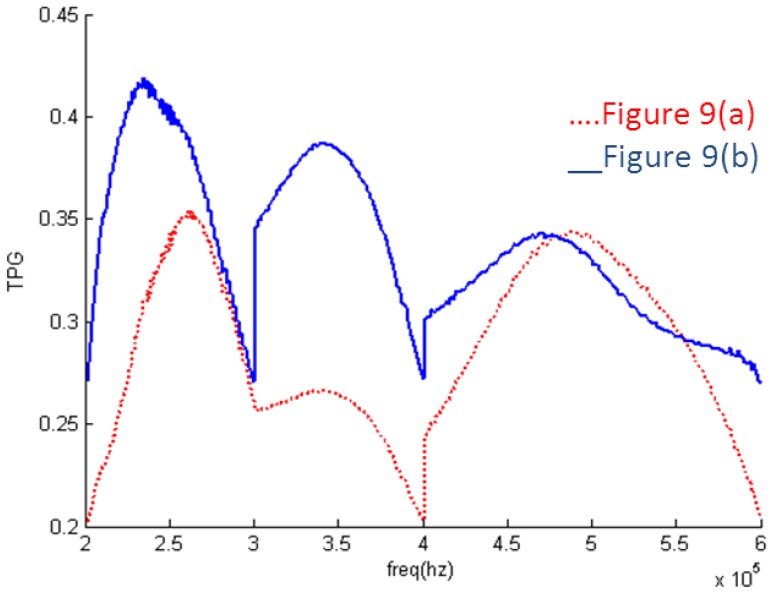
TPG of the EIMNs in Figure 9.

The test results are shown in Figure 8. As we can see, the impedance changes dramatically within the operating band due to the big difference in the reactance of different transducers. Based on the theory we introduced in Section 2 and the fact that the reactance of the transducer array is negative, an EIMN of T type with three branches is chosen to be the original structure. For comparison, two methods are used to design the EIMN. The topologies of two EIMNs are shown in Figure 9 and the comparison of their performance is shown in Figure 10. Although the EIMN designed by the algorithm introduced in this paper needs two more components than the EIMN of RFT, the performance is better at the most of the band.

**Figure 11.**
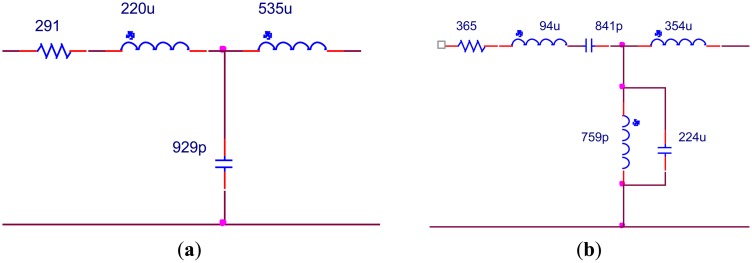
Results of two algorithms considering weight: (**a**) RFT; (**b**) algorithm introduced in Section 2.

**Figure 12.**
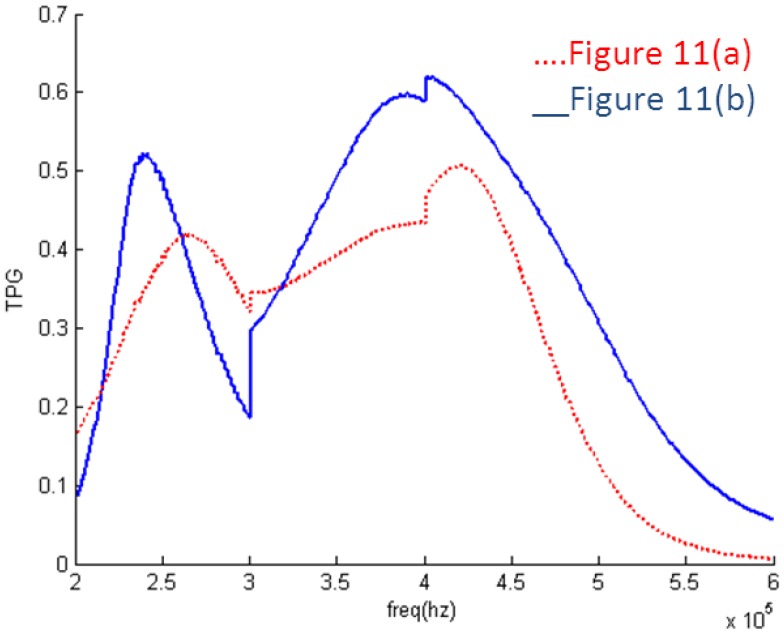
TPG of the EIMNs in Figure 11.

It can be seen in Figure 10 that the EIMN is badly affected by the drastic change of the impedance near 300 kHz and 400 kHz. To further improve the results, the actual application scenario of the transducer array needs to be considered. A transducer array is used in oil well logging. In practical use, the energy of the transmitted broadband signal concentrates around the central frequencies, 250 kHz, 350 kHz and 450 kHz. It is reasonable to reduce the weight of the frequency around 300 kHz and 400 kHz in the calculation. New results are shown in Figures 11 and 12. It is obvious that the performance of the new algorithm is still better and, as expected, the TPG gets better around the central frequencies of 250 kHz, 350 kHz and 450 kHz.

**Figure 13.**
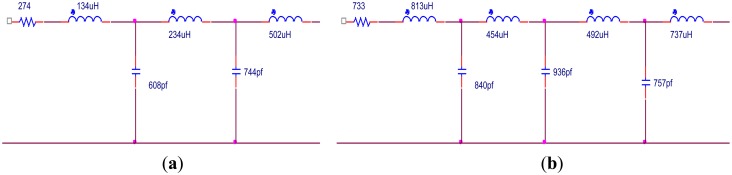
Topologies with five and seven branches: (**a**) five (**b**) seven.

**Figure 14.**
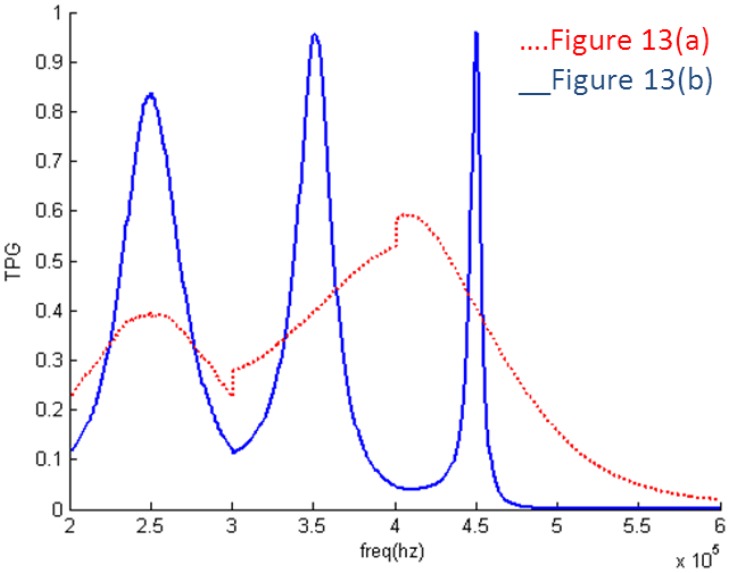
TPG of the EIMNs in Figure 13.

The results of the EIMNs with five and seven branches are also designed by RFT and shown in Figures 13 and 14. The results confirm the conclusion we mentioned in Section 2 that more branches do not mean better performance.

**Figure 15.**
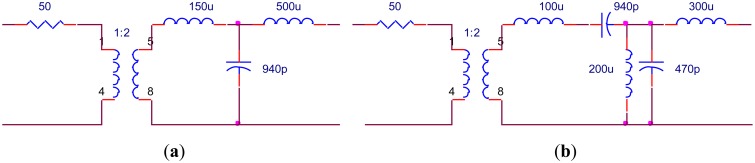
Topologies that the transformer turns ratio is 1:2 (**a**) RFT; (**b**) algorithm introduced in Section 2.

**Figure 16.**
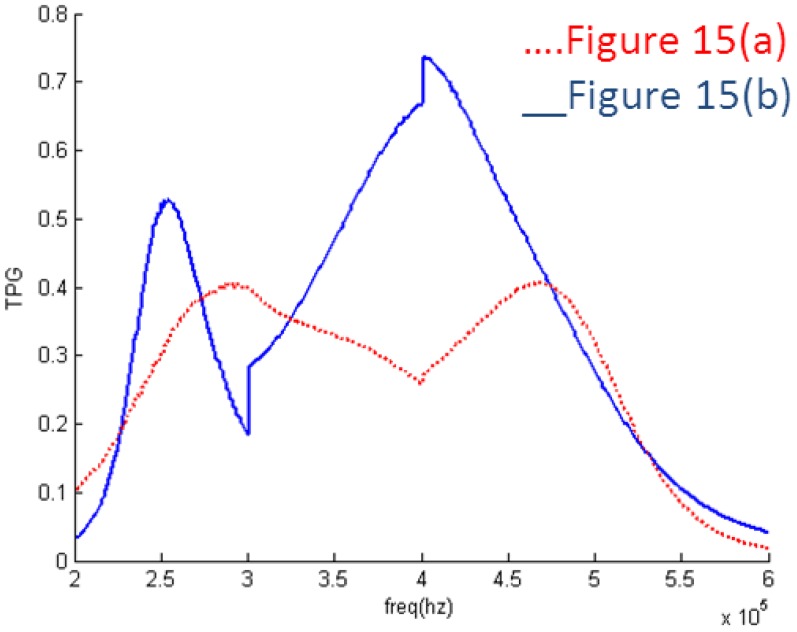
TPG of the EIMNs in Figure 15.

**Figure 17.**
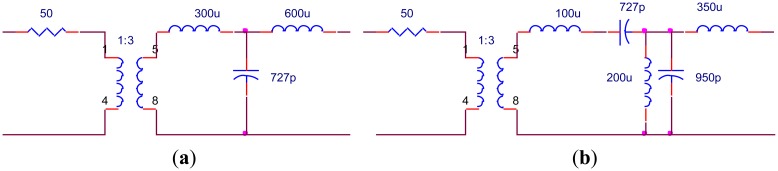
Topologies that the transformer turns ratio is 1:3 (**a**) RFT; (**b**) algorithm introduced in Section 2.

**Figure 18.**
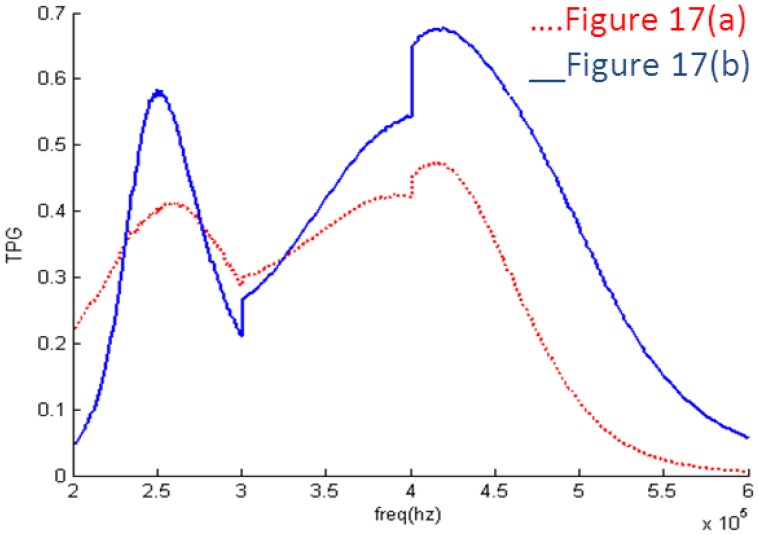
TPG of the EIMNs in Figure 17.

In order to verify the results, the values of the components have to be achievable. Based on the topologies shown in Figure 11, further optimization is adopted. During optimization the transformer turns ratio of 1:2 and 1:3 is fixed and other components are adjusted to the values that can be realized after optimization. The EIMNs where the transformer turns ratio is 1:2 are shown in Figure 15 and their TPGs are in Figure 16. The EIMNs where the transformer turns ratio is 1:3 are shown in Figure 17 and their TPGs are in Figure 18.

#### 3.2.2. Experiment and Results

An experiment is designed here to evaluate effect of the EIMN in increasing the power that the transducer consumes. When sinusoid signal is inputted, the calculation of the power the transducer consumes is shown as below. In Equation (4), V_L_ and I_L_ are the current and voltage of the transducer and the φ is the phase difference of the voltage and current:

(4) $$P_{L}=(V_{L}I_{L}/2)\cos\varphi$$

**Figure 19.**
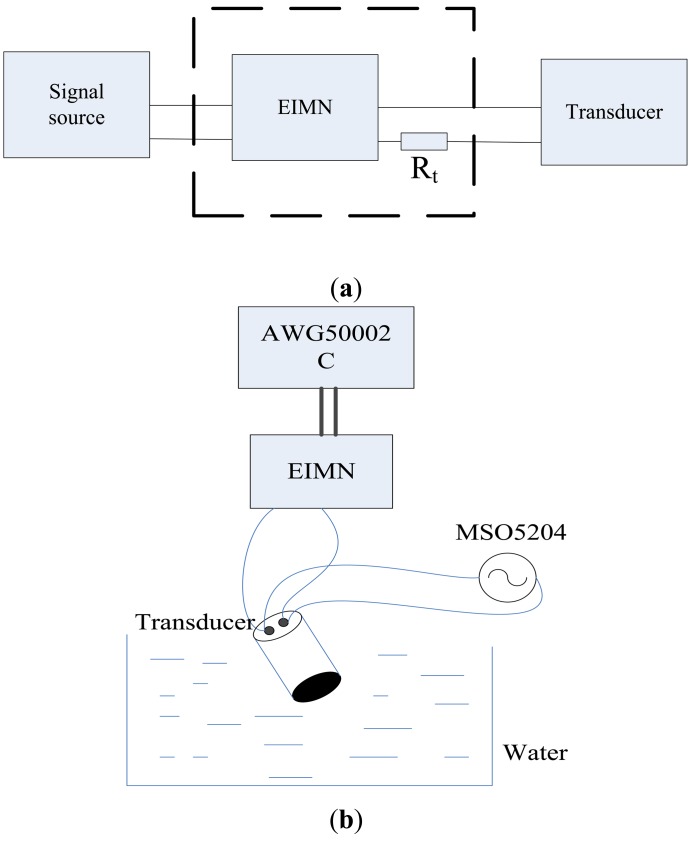
(**a**) A schematic block diagram for the experiment (**b**) the experimental setup.

The EIMN in Figure 17b is chosen to make an actual circuit to do the experiment. As is shown in Figure 19a, the EIMN connects both the signal source and the transducer. To facilitate measurement, a small resistance R_t_ is used to convert the current signal to a voltage signal.

During experiment, the transducer is put in water as shown in Figure 19b. A Tektronix AWG5002C waveform generator is used to generate sine wave and a Tektronix MSO5204 oscilloscope is used to measure and save data of V_L_ and I_L_. Parameter φ can be obtained by calculation.

**Figure 20.**
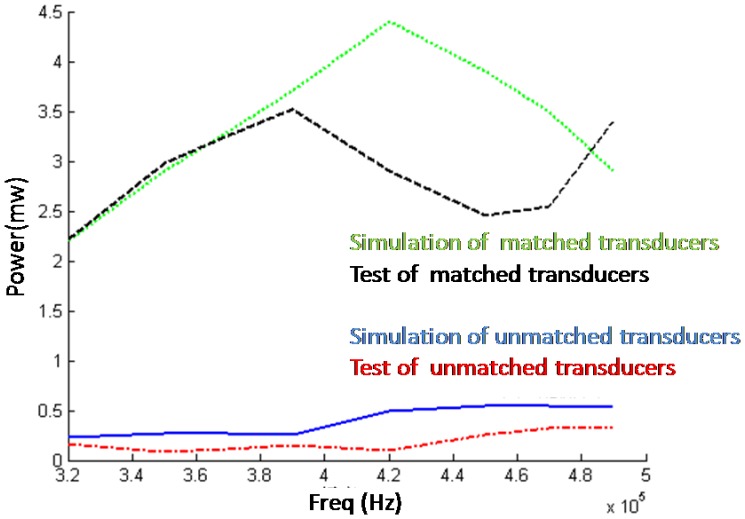
Comparison of experimental results and simulation results.

The results of experiment and simulation under both matched and unmatched conditions are shown in Figure 20. It is clear that the power that the transducers consume increase a lot when they are matched. Because of the high-frequency properties of the transformer, the energy above 400 KHz is largely filtered by the transformer. To improve the performance of the transformer, superpower soft ferrite that has good high-frequency performance might be helpful.

## 4. Conclusions

In this paper, an improved method of designing a broadband electrical impedance matching network for piezoelectric ultrasound transducera has been proposed. The method introduced here is based on a genetic algorithm. It can automatically synthesize the topology of electrical impedance matching network with resonant units. In order to decrease the computation time, results of popular methods are referenced to limit the size of the set of candidate topologies. Both BCGA and RCGA are implemented to optimize the topology and the components values, respectively. The simulation results have shown that the minimum of TPG obtained by our method based on GA is bigger than the topology with an LC ladder structure. Finally, an EIMN of an actual transducer array is designed and tested. Although more components might be used, simulation and experimental results have shown that the EIMN achieved by the improved method might have better performance.

## Figures and Tables

**Table 1.** Equalizer features for example.

**Parameter**	**RFT**	**RSE**	**GA before Simplex Algorithm**	**GA after Simplex Algorithm**
T_min_	0.849	0.855	0.8596	0.8611
Rs	2.2Ω	2.229Ω	2.184Ω	2.188Ω
Z1	0.352F	0.409F	0.443 F	0.430 F
Z2	2.909H	3.054H	3.067 H	3.018 H
Z3	0.922F	0.974F	1.014 F	1.002 F
